# The LSH/DDM1 Homolog MUS-30 Is Required for Genome Stability, but Not for DNA Methylation in *Neurospora crassa*

**DOI:** 10.1371/journal.pgen.1005790

**Published:** 2016-01-15

**Authors:** Evelina Y. Basenko, Masayuki Kamei, Lexiang Ji, Robert J. Schmitz, Zachary A. Lewis

**Affiliations:** 1 Department of Microbiology, University of Georgia, Athens, Georgia, United States of America; 2 Institute of Bioinformatics, University of Georgia, Athens, Georgia, United States of America; 3 Department of Genetics, University of Georgia, Athens, Georgia, United States of America; The University of North Carolina at Chapel Hill, UNITED STATES

## Abstract

LSH/DDM1 enzymes are required for DNA methylation in higher eukaryotes and have poorly defined roles in genome maintenance in yeast, plants, and animals. The filamentous fungus *Neurospora crassa* is a tractable system that encodes a single LSH/DDM1 homolog (NCU06306). We report that the Neurospora LSH/DDM1 enzyme is encoded by *mutagen sensitive-30* (*mus-30*), a locus identified in a genetic screen over 25 years ago. We show that MUS-30-deficient cells have normal DNA methylation, but are hypersensitive to DNA damaging agents. MUS-30 is a nuclear protein, consistent with its predicted role as a chromatin remodeling enzyme, and levels of MUS-30 are increased following DNA damage. MUS-30 co-purifies with Neurospora WDR76, a homolog of yeast Changed Mutation Rate-1 and mammalian WD40 repeat domain 76. Deletion of *wdr76* rescued DNA damage-hypersensitivity of Δ*mus-30* strains, demonstrating that the MUS-30-WDR76 interaction is functionally important. DNA damage-sensitivity of Δ*mus-30* is partially suppressed by deletion of *methyl adenine glycosylase-1*, a component of the base excision repair machinery (BER); however, the rate of BER is not affected in Δ*mus-30* strains. We found that MUS-30-deficient cells are not defective for DSB repair, and we observed a negative genetic interaction between Δ*mus-30* and Δ*mei-3*, the Neurospora RAD51 homolog required for homologous recombination. Together, our findings suggest that MUS-30, an LSH/DDM1 homolog, is required to prevent DNA damage arising from toxic base excision repair intermediates. Overall, our study provides important new information about the functions of the LSH/DDM1 family of enzymes.

## Author Summary

Inside cells, eukaryotic DNA exists in a highly packaged structure called chromatin. Chromatin packaging often inhibits enzymes that need to access the genetic information. It is therefore important for cells to regulate chromatin structure so that the genome can function properly. Mammalian LSH (Lymphoid-specific helicase; also known as HELLS, PASG, and SMARCA6) and *Arabidopsis* DDM1 (Decreased DNA methylation 1) are the founding members of the LSH/DDM1 subfamily of ATP-dependent chromatin remodelers. In mammals, the LSH enzyme is required for normal development, as well as oogenesis, spermatogenesis and T-lymphocyte proliferation. Similarly, the plant protein is required for development, and both proteins are important for regulating levels of DNA methylation, an important epigenetic mark. Recent studies suggest that LSH and DDM1 are also critical for genome integrity, but their precise functions are not understood. We have carried out genetic, genomic, and proteomic analyses to investigate an LSH/DDM1 homolog in a tractable model eukaryote, *Neurospora crassa*. We report that the Neurospora protein works in concert with the homologous recombination machinery to maintain genome stability. Our data provide important new information about the LSH/DDM1 family of enzymes.

## Introduction

Many chromatin-based processes require the activity of ATP-dependent chromatin remodeling enzymes, which can alter local chromatin structure by repositioning, removing, or restructuring nucleosomes [1–3]. Mammalian LSH (Lymphoid-specific helicase; also known as HELLS, PASG, and SMARCA6) and *Arabidopsis* DDM1 (Decreased DNA methylation 1) are the founding members of the LSH/DDM1 subfamily of ATP-dependent chromatin remodelers–one of 24 subfamilies that comprise the larger SNF2 enzyme family [4, 5]. In vitro, DDM1 is able to hydrolyze ATP and reposition nucleosomes on a short DNA template, demonstrating that the LSH/DDM1 subfamily includes *bona fide* chromatin remodeling enzymes [6]. Moreover, molecular and genetic studies have implicated LSH and DDM1 in a number of important cellular processes.

*Lsh* was originally identified as lymphocyte-specific; however, the gene is ubiquitously expressed in mammals [7–9]. In particular, high levels of *Lsh* are found in proliferating cells, suggesting that the protein might function during DNA synthesis or cell division. Subsequent studies revealed that *Lsh* is essential for development. Mice bearing homozygous deletions of *Lsh* die within 24 hours of birth, reportedly due to a host of developmental defects [8, 10]. Additional studies in chimeric mice or with tissue explants revealed that LSH is essential for both male and female meiosis [11, 12], as well as for proliferation of T-lymphocytes [13]. Thus, LSH is essential for gametogenesis and for proper development of the immune system. Notably, LSH has also been implicated in cancer [7, 14–18]. An in-frame Lsh deletion in the putative catalytic domain is frequently identified in human leukemias [7], and transplantation of hematopoietic precursors from *Lsh* ^-/-^ mice produced abnormal hematopoiesis and elevated rates of erythroleukemia in recipients [14]. Despite its role in these important processes, the molecular functions of LSH are not well understood.

*Lsh* mutant mice exhibit significantly reduced DNA methylation (5mC) at many sites in the genome [19–26]. Similarly, *Arabidopsis thaliana ddm1* mutants display reduced DNA methylation and developmental defects, suggesting that at least some LSH/DDM1 functions are conserved across eukaryotic kingdoms [27–33]. Recently, studies in both plants and animals have uncovered a role for LSH/DDM1 in maintenance of genome stability. *Arabidopsis* DDM1-deficient mutants are hypersensitive to a variety of DNA damaging agents, including MMS (methyl methanesulfonate) [34, 35]. Similarly, mammalian *Lsh*^*-/-*^ cells are hypersensitive to DNA damage and are unable to mount a robust DNA damage response [36]. There is some controversy regarding the relationship between the DNA methylation and DNA damage phenotypes of LSH/DDM1-deficient cells. The DNA damage-sensitivity phenotype of *ddm1* plants was proposed to be an indirect effect of DNA hypomethylation [35], whereas in animals, stable knockdown of *Lsh* in immortalized lung fibroblasts led to hypersensitivity to DNA damage before a reduction in DNA methylation levels was observed [36]. Notably, an LSH homolog was also implicated in genome maintenance in *Saccharomyces cerevisiae*, an organism that lacks DNA methylation. The yeast gene, named *IRC5* (Increased repair centers-5), was uncovered in a high throughput screen for deletion strains that accumulate spontaneous DNA repair foci [37]. Thus, LSH-family enzymes are important for genome stability in fungi, plants, and animals, but precisely how LSH/DDM1 homologs control DNA methylation or genome stability is not clear.

LSH-family members are absent from several model systems including *Drosophila melanogaster*, *Caenorhabditis elegans*, and *Schizosaccharomyces pombe* [5], but the model fungus *Neurospora crassa* encodes a single LSH/DDM1 homolog (NCU06306; also called Chromatin Remodeling Factor 5) [5, 38]. *N*. *crassa* is a particularly attractive model for studies of chromatin structure and function because its complement of chromatin modifications and chromatin-associated proteins is similar to higher eukaryotes. For example, hallmarks of heterochromatin such as histone H3 lysine-9 methylation (H3K9me3), Heterochromatin protein-1 and DNA methylation are shared between Neurospora and higher eukaryotes, but are all absent from *S*. *cerevisiae* [39–42]. To gain insights into the functions of the LSH/DDM1 subfamily, we performed molecular, genetic and genomic analyses to investigate *N*. *crassa* NCU06306/CRF5. We found that this LSH/DDM1 homolog is not required for DNA methylation, but is essential for survival from DNA damage. *ncu06306/crf5-1* is allelic to the previously described *mutagen sensitive-30* (*mus-30*). The encoded protein is localized to the nucleus and interacts with WDR76, a conserved WD40 domain-containing protein. Based on genetic interactions with known DNA repair components, we propose that the Neurospora LSH/DDM1 homolog functions to limit genome instability resulting from toxic base excision repair intermediates.

## Results

### The LSH/DDM1 homolog is not required for DNA methylation in *N*. *crassa*

**Fig 1:**
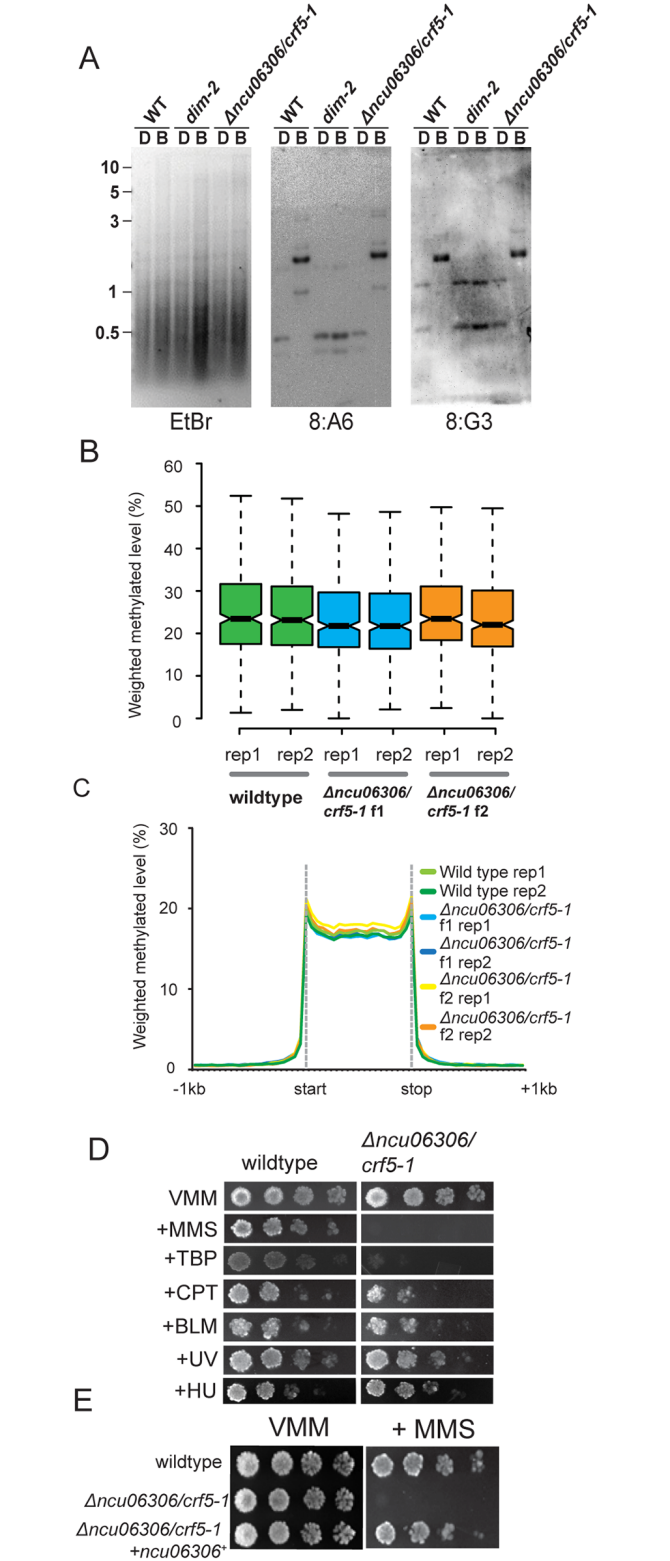
Neurospora cells lacking the LSH/DDM1 homolog have normal DNA methylation but are hypersensitive to DNA damage. (A) Southern hybridizations with probes corresponding to the methylated 8:A6 and 8:G3 regions were performed using genomic DNA from the indicated strains digested with the cytosine-methylation-sensitive BfuCI [B] and–insensitive DpnII [D] restriction enzymes. The gel stained with Ethidium Bromide is also shown (EtBr). Numbers on the left correspond to the DNA ladder (kb). (B) Box plots showing the average methylation level for all methylated regions in wildtype, plus Δ*ncu06306/crf5-1* strains from f1 and f2 generations. Two biological replicates are shown for each strain. The notches indicate the 95% confidence interval around the median. Overlapping notches indicate that the samples are not statistically different. (C) A metaplot showing the average distribution of DNA methylation across all methylated domains for wildtype and for Δ*ncu06306/crf5-1* strains from f1 and f2 generations. (D) Serial dilutions of conidia (10^4^−10^1^) of wildtype and *Δncu06306/crf5-1* were spotted on Vogel’s Minimal Medium (VMM) with or without the indicated genotoxic agents: methyl methanesulfonate (MMS; 0.025%), tert-Butyl hydroperoxide (TBP; 100 μM), camptothecin (CPT; 0.3 μg/mL), Bleomycin (BM; 0.2 μg/mL), Hydroxyurea (HU; 7mM). Cells were also exposed to Ultraviolet light (UV; 300 x 100mJ/cm2). (E) Introduction of wildtype *ncu06306/crf5-1+* complements the MMS-hypersensitive phenotype of *Δncu06306/crf5-1*. Serial dilutions of the indicated strains were spotted on minimal medium (VMM) with or without 0.025% MMS.

Neurospora encodes a single LSH/DDM1 homolog encoded by NCU06306 and given the name Chromatin Remodeling Factor 5 (CRF5) based on its predicted coding sequence [38]. Like LSH and DDM1, NCU06306/CRF5 contains a characteristic SNF2 motor domain made up of an N-terminal SNF2_N DEAD box helicase domain and a C-terminal HelicC domain, but lacks other conserved domains. To determine if NCU06306/CRF5 is important for DNA methylation in *N*. *crassa*, we first performed Southern blot analysis to examine DNA methylation levels at two well-studied methylated regions (8:A6 and 8:G3) [43]. DNA methylation levels were similar to wildtype at both regions (Fig 1A). In plant *ddm1* mutants, loss of DNA methylation is gradual; 5mC levels progressively decline when *ddm1* homozygous mutants are inbred for multiple generations [27]. We therefore performed MethylC-seq to examine genome-wide DNA methylation levels in f1 and f2 progeny derived from homozygous crosses of *Δncu06306/crf5-1* parents. We note that NCU06306/CRF5 was not required for meiosis, in contrast to mammalian LSH. As controls, we performed methylC-seq for wildtype and *dim-2* (*defective in methylation-2*), which lacks DNA methylation [44]. We identified methylated regions in Neurospora by calling differentially methylated regions (DMR) between wildtype and a fully unmethylated genome (generated *in silico;* see Methods), and we calculated the average weighted methylation level for all 5mC regions in wildtype and *Δncu06306/crf5-1* isolates from f1 and f2 generations (Fig 1B and S1 Table). Average methylation levels in the *Δncu06306/crf5-1 i*solates were not statistically different from wildtype. We next constructed metaplots to examine the distribution of 5mC across all methylated regions for wildtype and *Δncu06306/crf5-1 i*solates from f1 and f2 generations (Fig 1C). The methylation profile of the *Δncu06306/crf5-1* strain was similar to wildtype for both strains. These data suggest that NCU06306/CRF5 does not control the levels or the distribution of 5mC within normally methylated regions, in contrast to LSH/DDM1 enzymes in higher eukaryotes (S1 Table). Finally, we used DMR analysis to compare 5mC regions in wildtype and *Δncu06306/crf5-1* strains. We identified twenty-two regions with subtle changes in the level of methylation in one or more *Δncu06306/crf5-1* isolates. However, these subtle differences likely represent sequence polymorphisms between the *mat A* reference strain and the *mat a* strain (S2 Table). Together, these data demonstrate that NCU06306/CRF5 is not required for normal DNA methylation in *N*. *crassa*.

### The LSH/DDM1 homolog is essential for DNA damage tolerance

We next asked if *Δncu06306/crf5-1* is required for survival from DNA damage, as proposed for other LSH/DDM1 homologs. We examined growth of *Δncu06306/crf5-1* in the presence of several DNA replication and DNA repair inhibitors (Fig 1D). *Δncu06306/crf5-1* cells were not hypersensitive to UV light or Hydroxyurea, which inhibits ribonucleotide reductase [45]. Similarly, *Δncu06306/crf5-1* cells displayed wildtype resistance to Bleomycin, which is thought trigger double strand breaks [46], and only displayed limited sensitivity to the topoisomerase I inhibitor camptothecin (CPT) [47]. In contrast, *Δncu06306/crf5-1* cells were unable to grow on medium containing methyl methanesulfonate (MMS; 0.025%), which can collapse replication forks, leading to double strand breaks [48, 49]. *Δncu06306/crf5-1* were also hypersensitive to oxidative damage by tert-Butyl hydroperoxide [50].

Knockout strains have been shown to accumulate second-site mutations [51]. To confirm that the DNA damage-hypersensitive phenotype of *Δncu06306/crf5-1* is caused by deletion of the *ncu06306/crf5-1* gene (NCU06306), we introduced a wildtype copy of *ncu06306/crf5-1+* into the deletion strain and tested for growth on MMS. Wildtype *ncu06306/crf5-1+* restored growth, confirming that *ncu06306/crf5-1* is required for survival from MMS-induced DNA damage (Fig 1E).

### *crf5* is allelic with *mus-30*

**Fig 2:**
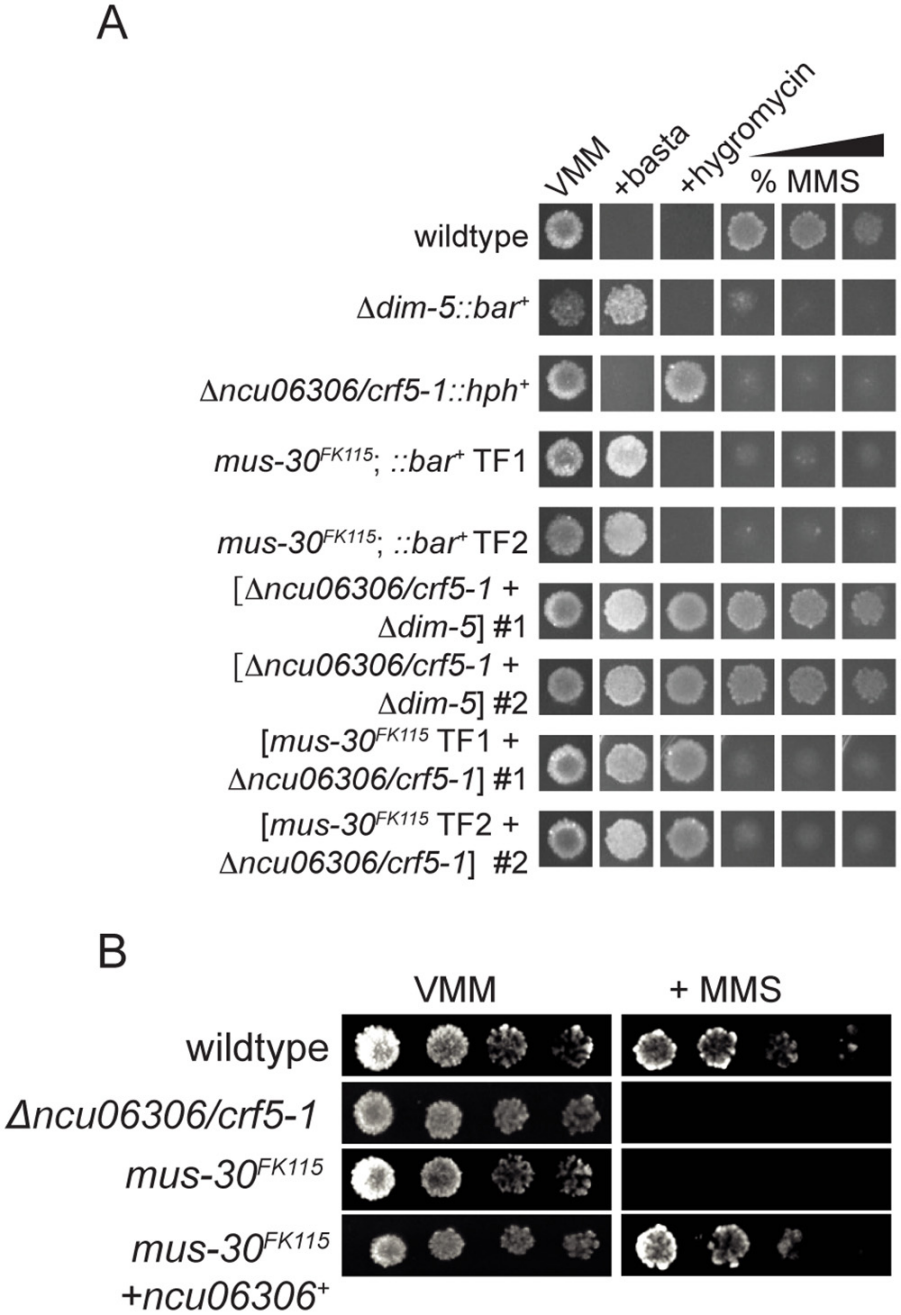
*ncu06306/crf5-1* is allelic to *mutagen sensitive-30*. (A) Conidia from the indicated strains or heterokaryons were spot tested on minimal medium (VMM) with or without basta, hygromycin, or increasing concentrations of MMS (0.010%, 0.015%, 0.025%). By convention, heterokaryons are indicated with brackets enclosing the genotypes of contributing nuclei. (B) Introduction of wildtype *ncu06306/crf5-1*^*+*^ complements the MMS-hypersensitive phenotype of *mus-30*^*FK115*^. Serial dilutions of conidia (10^4^−10^1^) of wildtype, *Δncu06306/crf5-1*, or the complemented strain were spotted on Vogel’s Minimal Medium (VMM) with or without MMS.

A previous screen for mutagen sensitive strains led to the identification of *mutagen-senstive-30* (*mus-30*), which had been mapped to a region on LGIV that includes the *ncu06306/crf5-1* gene [52]. Like *Δncu06306/crf5-1*, *mus-30* ^*FK115*^ is sensitive to MMS, but not to HU or UV light [52]. To test the possibility that *ncu06306/crf5-1* and *mus-30* are allelic, we sequenced the *ncu06306/crf5-1* gene from the original *mus-30*^*FK115*^ isolate. The *mus-30* ^*FK115*^ strain contains a single base change in the *ncu06306/crf5-1* locus, which is predicted to produce an Arginine to Proline substitution at position 809. This mutation is within the predicted HelicC domain [53]. We next tested for complementation in heterokaryons of *Δncu06306/crf5-1* and *mus-30*^*FK115*^. The *mus-30* ^*FK115*^ strain was transformed with a basta-resistance cassette to allow construction of forced heterokaryons. Six heterokaryons of *mus-30*^*FK115*^;:: *bar*+ and *Δncu06306/crf5-1*::*hph*^*+*^ were generated from individual basta-resistant transformants and maintained on medium containing both hygromycin and basta. To test for complementation, condia were spotted on medium containing hygromycin, basta, or MMS (0.015%, 0.020%, and 0.025%). Control heterokaryons were constructed by mixing conidia of the basta-resistant, MMS-sensitive Δ*dim-5* strain with conidia from the hygromycin-resistant, MMS-sensitive *Δncu06306/crf5-1* strain. Representative heterokaryons are shown in Fig 2A. All control heterokaryons [*Δncu06306/crf5-1*::*hph*^*+*^ *+ Δdim-5*::*bar*^+^] were able to grow on medium containing MMS, demonstrating that the MMS-sensitivity phenotypes of *Δncu06306/crf5-1* and *Δdim-5* strains are recessive. In contrast, none of the heterokaryons of *mus-30* ^*FK115*^ and *Δncu06306/crf5-1* were able to grow on medium containing MMS (six independent heterokaryons were tested), suggesting that *ncu06306/crf5-1* and *mus-30* are allelic.

To confirm this, we introduced a wildtype copy of the *ncu06306/crf5-1* gene into the *mus-30* ^*FK115*^ strain by co-transformation with a basta-resistance cassette. A fraction of basta-resistant transformants are expected to integrate the *ncu06306/crf5-1* sequence along with the basta-resistance cassette. Of 40 basta-resistant transformants tested, 15 displayed robust growth in the presence of MMS (Fig 2B and S1 Fig). No MMS-resistant transformants were obtained when *mus-30* ^*FK115*^ was transformed with the basta*-*resistance cassette alone. Together, these data demonstrate that *ncu06306/crf5-1* is allelic to *mus-30*. We hereafter refer to NCU06306/CRF5 as MUS-30.

### MUS-30 has nuclear localization and is induced by DNA damage

**Fig 3:**
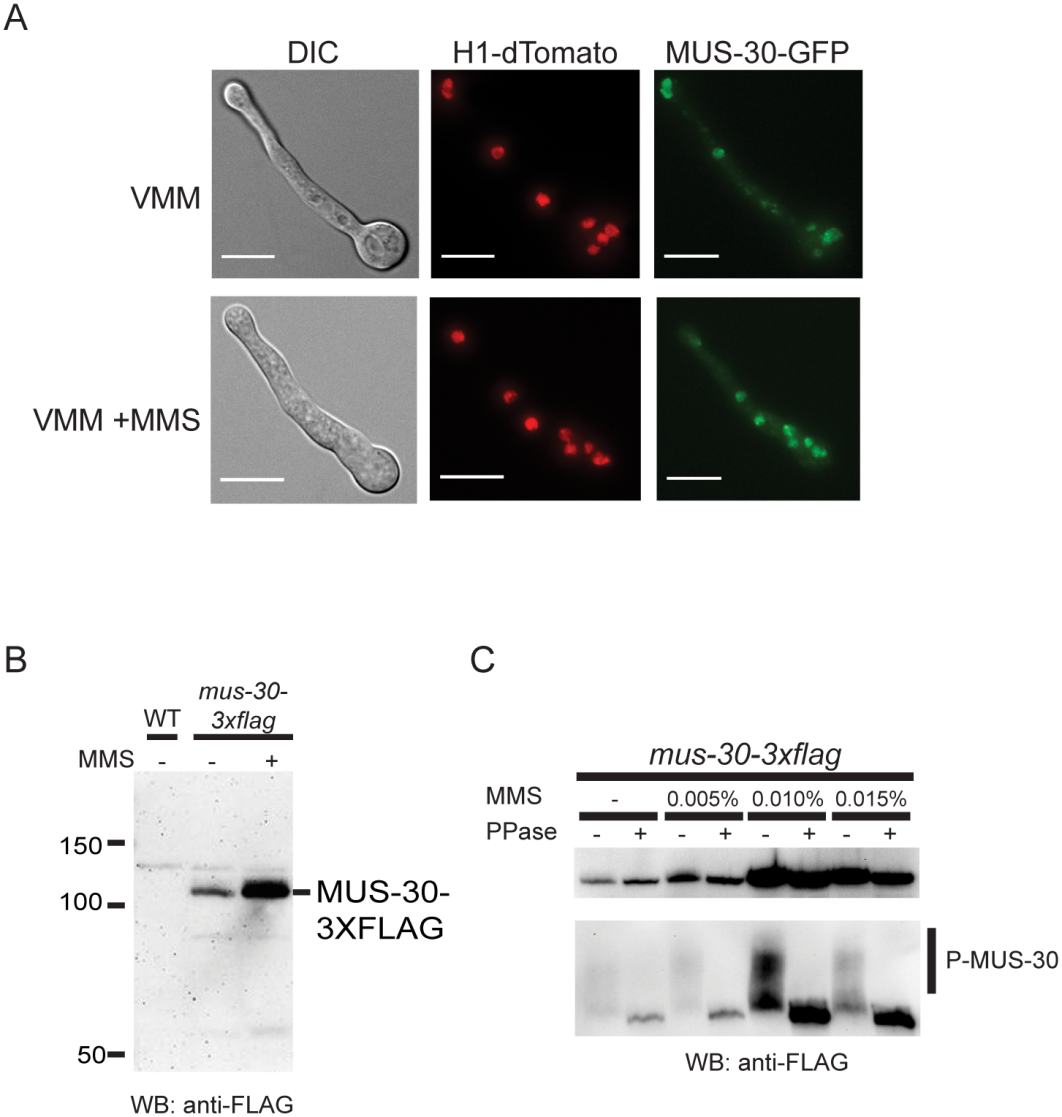
MUS-30 is a nuclear phosphorylated protein that is induced by DNA damage. (A) MUS-30-GFP and H1-dTomato were visualized in live cells grown for 5 hours in minimal medium (VMM; top) or for 2 hours in VMM followed by 3 hours in VMM+0.015% MMS (bottom). The scale bar indicates 6 μm (B) Protein extracts from a wildtype strain and a *mus-30-3xflag* strain subjected to Western blotting using anti-FLAG antibodies. The *mus-30-3xflag* strain was grown in the presence or absence of MMS, as indicated. (C) Protein extracts were isolated from *mus-30-3xflag* strains grown overnight in minimal medium with or without MMS. Total protein extracts were incubated with or without lambda phosphatase (+ or—PPase) and resolved by SDS-PAGE (top panel) or the FLAG immunoprecipitate fraction was resolved on a Phos-tag gel (bottom panel), transferred to a membrane, and probed with anti-FLAG antibodies. Phosphorylated MUS-30-3XFLAG is evident as a low mobility smear (P-MUS-30).

MUS-30 is predicted to function as a chromatin remodeler and is therefore expected to localize to the nucleus. To test this, we constructed a GFP-tagged version of MUS-30 using a standard “knock-in” approach [54]. GFP coding sequence was integrated by homologous recombination into the 3’ end of the *mus-30* gene. Primary transformants were backcrossed to isolate homokaryons and individual *mus-30-gfp* strains were tested for growth on MMS to confirm that the GFP fusion construct was functional (S2 Fig). In live cells, MUS-30-GFP was localized to the nucleus, consistent with its predicted role as a chromatin remodeling enzyme (Fig 3A). Some DNA repair proteins alter their localization in response to DNA damage. We treated cells with MMS for three hours and then examined the localization patterns of MUS-30-GFP before, during, and after MMS treatment. A diffuse nuclear localization pattern was observed in the presence and absence of MMS. However, we detected an increase in overall fluorescence in some experiments, suggesting that MUS-30 protein levels may be increased in response to DNA damage (Fig 3A). To determine if MUS-30 protein levels are increased in MMS treated cells, we constructed a FLAG-tagged version of MUS-30 and performed Western blot analysis. The *mus-30-3xflag* strain was able to grow on MMS, indicating that the tagged version of the protein was functional (S2 Fig). Total protein isolated from wildtype and the *mus-30-3xflag* strain grown in minimal medium and subjected to Western blotting with anti-flag antibodies. We detected an ~106kD protein in extracts from the *mus-30-3xflag* strain, consistent with the predicted size of the MUS-30-3XFLAG fusion protein (Fig 3B). We next compared the level of MUS-30-3XFLAG expression in minimal medium and in the presence of MMS. MUS-30-3XFLAG levels were higher in MMS-containing medium. Under certain gel conditions, the FLAG antibody detected two bands, raising the possibility that MUS-30 is post-translationally modified. Phosphorylation of proteins is often associated with signaling in response to DNA damage [55]. To determine if MUS-30 is phosphorylated, we resolved protein extracts from the *mus-30-3xflag* strain on a Phos-Tag gel, which reduces the mobility of phosphorylated proteins (Fig 3C) [56]. We observed a shift in mobility of MUS-30-3XFLAG under all conditions examined. Treating extracts with lambda phosphatase eliminated the slower migrating form of the protein, suggesting that MUS-30-3XFLAG is indeed phosphorylated but that phosphorylation does not occur specifically in response to DNA damage.

### *mus-30* interacts genetically with *mag-1* and *mei-3*

**Fig 4:**
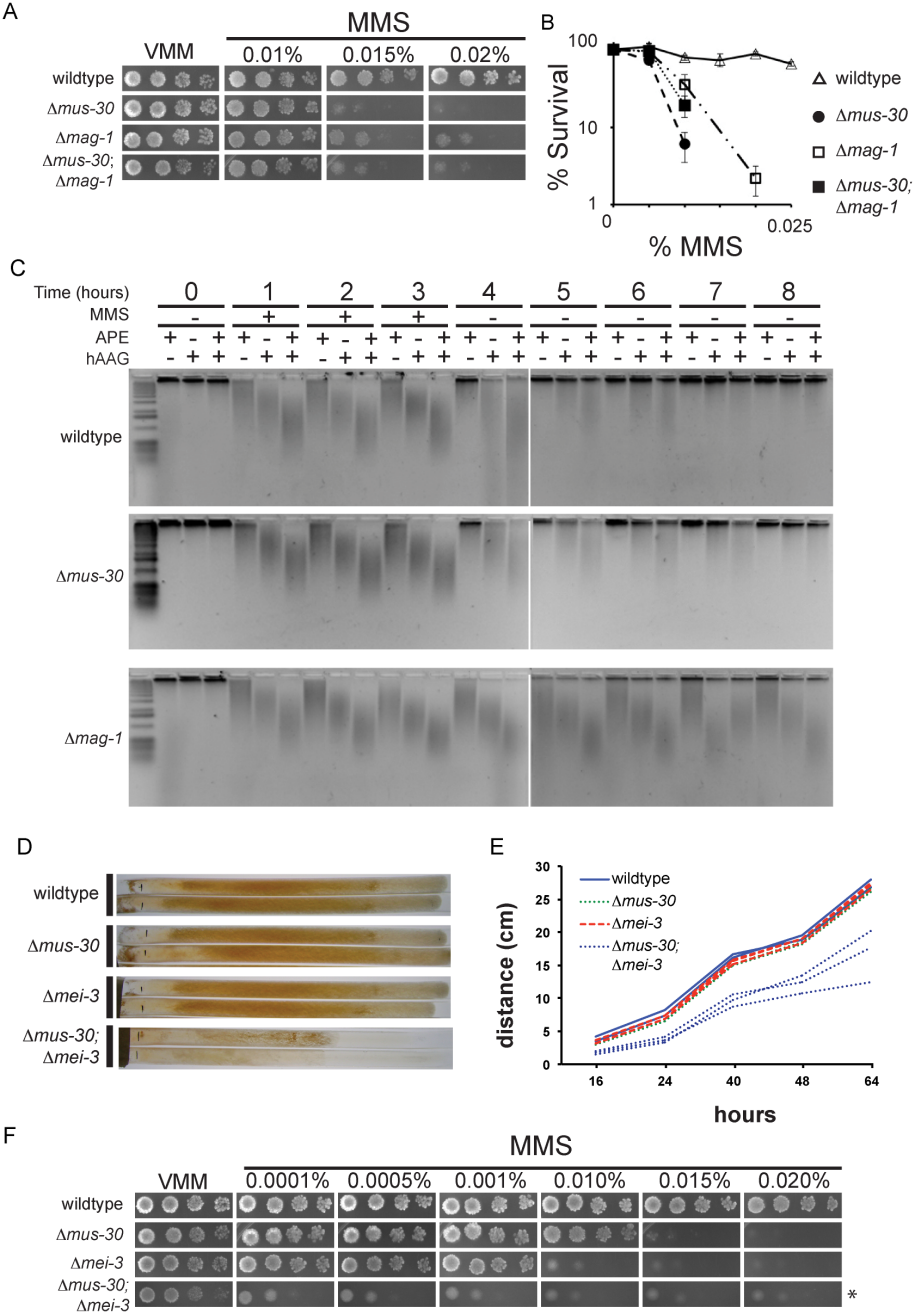
*Δmus-30* interacts genetically with *Δmag-1 and Δmei-3*. (A) Serial dilutions of conidia (10^4^−10^1^) were spot tested on minimal medium (VMM) with or without the indicated concentrations of MMS for the indicated strains. (B) The average number of colonies for each genotype is shown for the indicated concentrations of MMS. For each concentration, % survival is shown relative to no MMS control. At least two isolates of each genotype were analyzed. Error bars show the standard deviation. (C) Repair of MMS-induced damage is shown in wildtype, *Δmus-30*, and *Δmag-1* cells, as indicated. Genomic DNA was isolated from cells before, during, and after MMS exposure, as indicated. DNA was treated with Human Alkyladenine DNA Glycosylase (hAAG), apurinic/apyrimidinic endonuclease (APE), or both to induce ssDNA breaks at methylated bases or abasic sites. The size of ssDNA was visualized at each time point by alkaline electrophoresis. A low molecular weight smear indicates the presence of unrepaired DNA after MMS treatment. (D) Images of race tubes containing minimal medium show the relative growth rates of the indicated strains. (E) The linear growth rate is plotted for multiple isolates of each genotype shown in D. (F) Serial dilutions of conidia (10^4^−10^1^) were spotted on minimal medium (VMM) with or without the indicated concentrations of MMS for wildtype and the indicated single mutants. For *Δmei-3*; *Δmus-30* strains, a dilution series from 10^5^−10^2^ was used due to poor spore viability (asterisk).

Genetic interactions can provide insights into gene function. Positive genetic interactions often indicate that the products of the interacting genes function in the same pathway, whereas negative interactions suggest that two gene products perform compensatory functions in separate pathways [57]. Positive genetic interactions occur when the fitness of a double mutant is better than the expected phenotype. For example, combining two mutations that cause MMS-sensitivity is expected to produce a double mutant that has a higher level of MMS sensitivity than either single mutant. In contrast, a negative genetic interaction occurs when the fitness of the double mutant is worse than the phenotype expected from combining the two single mutant phenotypes. Genetic interaction analysis has been used extensively to place Neurospora DNA repair mutants into epistasis groups [58]. For example, members of the *uvs-6* epistasis group exhibit positive genetic interactions with one another and encode components of the homologous recombination repair pathway [59]. *Δmus-30* strains are highly sensitive to MMS, which generates methylated bases that can stall replication forks and indirectly lead to DSBs [48, 49, 60–63]. The primary mechanism for repair of MMS-induced damage is base excision repair (BER). We therefore crossed *Δmus-30* to *Δmag-1*, a putative BER glycosylase that removes methyl-adenine bases generated by MMS [38], and we determined the level of MMS sensitivity in wildtype, single mutant, and double mutant progeny. We observed a positive genetic interaction between *Δmus-30* and *Δmag-1* (Fig 4A and 4B). The *Δmus-30*; *Δmag-1* double mutant was more tolerant to MMS than *Δmus-30* single mutants, exhibiting a level of sensitivity that was similar to the *Δmag-1* strain. Consistent with its predicted role as a methyl-adenine glycosylase, *Δmag-1* did not suppress the TBH-hypersensitive phenotype of *Δmus-30* strains (S3 Fig). Taken together, these data could indicate that MUS-30 functions in the BER pathway downstream of base removal. However, it has been shown that BER intermediates are themselves mutagenic [49, 62–65]. Therefore, these data could also indicate that MUS-30 is required to prevent or repair DNA damage that arises when replication forks encounter toxic BER intermediates.

To distinguish between these possibilities, we asked if *Δmus-30* was able to repair alkylated bases at a rate that was similar to wildtype. Cells were exposed to MMS and harvested during and after MMS exposure. To monitor repair of MMS-induced damage, genomic DNA was treated with recombinant BER enzymes to generate single-strand breaks at MMS-damaged bases and abasic sites [66]. The DNA was then resolved by alkaline gel electrophoresis to examine kinetics of repair; unrepaired DNA that contained alkylated bases or abasic sites runs as a low molecular weight smear. As expected, *Δmag-1* failed to repair MMS-damaged bases, consistent with its predicted role as a BER glycosylase. Both wildtype and *Δmus-30* were able to remove and repair alkylated bases with similar kinetics (Fig 4C). These data demonstrate that MUS-30 is not required for BER, but is likely important for preventing or repairing DNA damage that results from toxic BER intermediates.

It was previously reported that mammalian LSH was required for efficient double strand break repair (DSB) [36]. We asked if MUS-30 influenced how double-strand breaks are repaired in *N*. *crassa* using an established transformation assay. Ectopic DNA sequences are inserted into the *N*. *crassa* genome via the homologous recombination (HR) or non-homologous end joining (NHEJ) DSB repair pathways [67, 68]. We transformed wildtype or *Δmus-30* strains with a basta-resistance cassette flanked by 5’ and 3’ sequences corresponding to the *methyltryptophan resistance* (*mtr*) locus. Cells that undergo cassette integration by HR are resistant to basta and to Fluorophenylalanine (FPA), whereas cells that undergo non-homologous integration are resistant to basta, but not FPA [67]. As expected, cassette integration occurred exclusively by HR in *Δmus-52* or *Δmus-53*, which lack required NHEJ components, while no HR events occurred in a *Δmei-3* control strain. *mei-3* encodes the *N*. *crassa* homolog of yeast RAD51 and is required for DSB repair via homologous recombination (HR) [67, 69]. The frequency of homologous integration in the *Δmus-30* strain was similar to wildtype, suggesting that MUS-30 is not required for HR or for NHEJ in Neurospora (Table 1). Furthermore, the transformation efficiency was similar in all strains tested, with the exception of Δ*mus-53*, which showed a reduction in transformation efficiency as reported previously [67]. These data demonstrate that MUS-30 is not required for general DSB repair in *N*. *crassa*.

**Table 1:** Efficiency of DSB repair.

Genotype	Transformation Frequency^1^	Frequency of HR events^2^
wildtype	1.19 ± 0.78 x 10^−7^	15%
Δ*mei-3*	0.75 ± 0.04 x 10^−7^	0%
Δ*mus-53*	0.08 ± 0.06 x 10−^7^	100%
Δ*mus-52*	0.92 ± 0.98 x 10−^7^	100%
Δ*mus-30*	1.43 ± 0.46 x 10−^7^	15%
Δ*wdr76*	1.07 ± 0.03 x 10^−7^	10%
Δ*wdr76*; Δ*mus-30*	1.12 ± 0.01 x 10^−7^	15%

^1^ The number of Basta-resistant transformants per total cell number. Values are the averaged from three independent experiments.

^2^ The percentage of Basta-resistant transformants that were also FPA-resistant. 20 transformants were tested for each strain.

MMS-induced damage and BER intermediates can lead to collapsed replication forks that can be restarted in a RAD51-depdendent manner [49, 60–65, 70–73]. To test if MUS-30 prevents replication fork collapse or facilitates MEI-3-dependent replication fork restart, we tested for genetic interactions between Δ*mus-30* and Δ*mei-3*. We reasoned that a positive genetic interaction would suggest that MUS-30 facilitates MEI-3 dependent replication fork restart, whereas a negative genetic interaction could suggest MUS-30 is required to prevent collapsed forks at MMS-damaged bases and BER intermediates. We observed a striking synthetic growth defect for Δ*mus-30*; Δ*mei-3* double mutants, which was evident even in the absence of exogenous DNA damaging agents. We performed race tube analysis with multiple isolates of each genotype to quantify the linear growth rate. The growth rates of wildtype, *Δmus-30*, and *Δmei-3* were similar, whereas all isolates of *Δmus-30*; *Δmei-3* double mutants displayed markedly slower growth (Fig 4D and 4E). We next tested the level of MMS-sensitivity for each genotype **(**Fig 4F). *Δmus-30* and *Δmei-3* single mutants were unable to grow in the presence of 0.015% and 0.010% MMS, respectively, while *Δmus-30; Δmei-3* conidia failed to grow on the lowest MMS concentration tested (0.0001%). Thus, MEI-3 is critical for repairing DNA damage that accumulates in the *Δmus-30* mutant strain. Together, these data demonstrate that MUS-30 is not generally required for DSB repair and suggest that MUS-30 is important for preventing DNA damage that arises from toxic base excision repair intermediates.

### Identification of a MUS-30 binding partner

**Fig 5:**
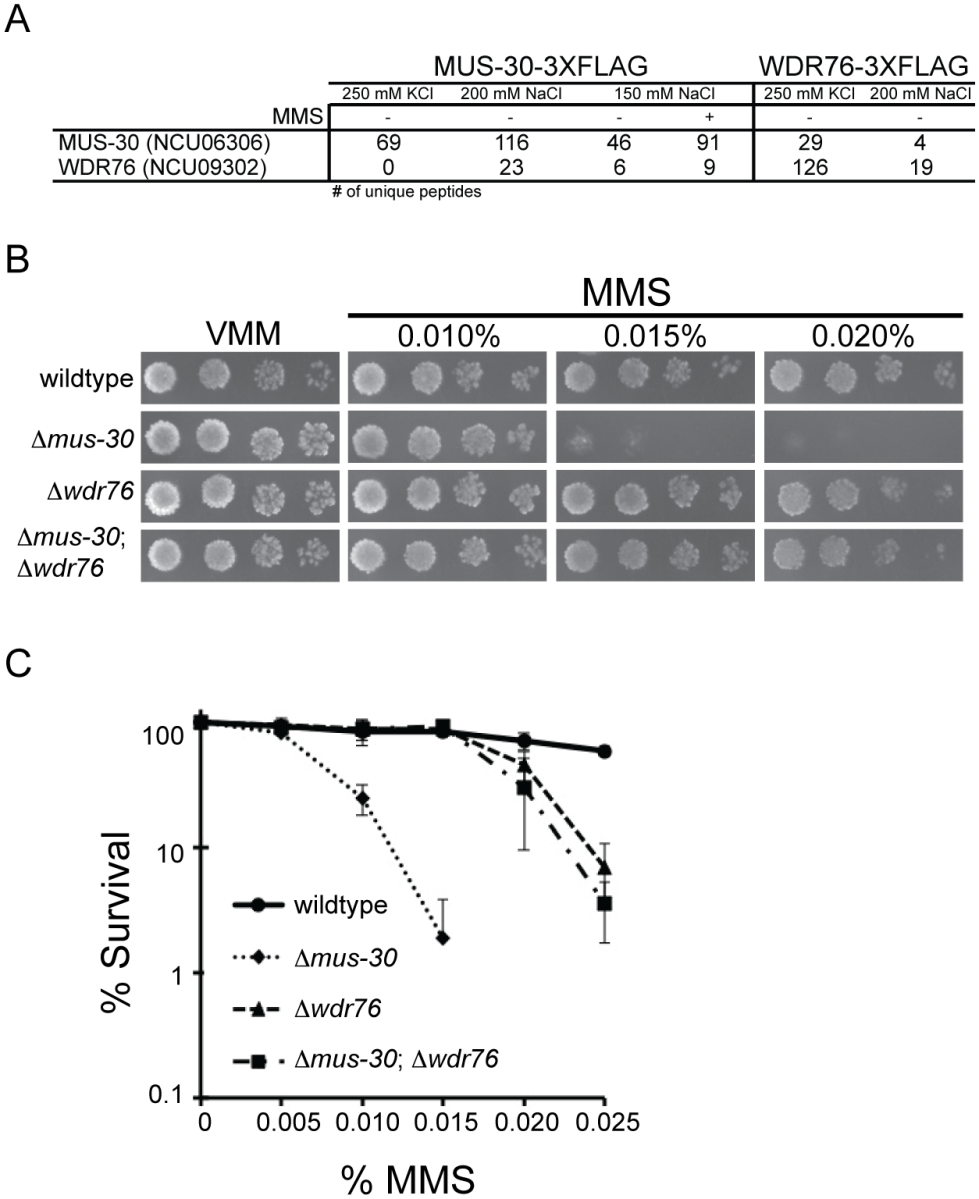
WDR76 is a MUS-30 interacting protein. (A) The table indicates the number of unique peptides corresponding to MUS-30 (NCU6306) or WDR76 (NCU09302) for the indicated purification conditions. (B) Serial dilutions of conidia from the indicated strains (10^4^−10^1^) were spot tested on minimal medium (VMM) with or without the indicated concentrations of MMS. (C) The average number of colonies is shown for the indicated concentrations of MMS for wildtype, *Δmus-30*, *Δwdr-76*, and *Δmus-30; Δwdr76* double mutants. For each concentration, % survival is shown relative to no MMS control. At least two isolates of each genotype were analyzed. Error bars show the standard deviation.

Many chromatin remodeling proteins exist in multi-subunit complexes. To gain insights into the biochemical function of MUS-30, we sought to identify MUS-30-interacting proteins using a proteomics approach. We used antibodies that recognize the FLAG epitope to purify MUS-30-3XFLAG and identified co-purified proteins by mass spectrometry. As negative controls, we performed a mock purification from the wildtype strain, which does not express a FLAG-tagged protein, and we performed purifications of two components of the previously described DCDC complex, DIM-5-3XFLAG and DIM-9-3XFLAG [74]. To eliminate background hits from our list of putative MUS-30-interacting proteins, we removed proteins that were identified in the “mock” sample (no FLAG-tagged protein), the DIM-5-3XFLAG sample, or DIM-9-3XFLAG sample, and we removed proteins that were identified by a single unique peptide hit (*i*.*e*. only proteins identified by two or more unique peptides passed the filter). Purification of MUS-30-3XFLAG in buffer containing 250 mM KCl failed to identify any specific interacting proteins, suggesting that MUS-30 does not exist in a stable multi-subunit complex. However, purification of MUS-30-3XFLAG from protein extracts made with buffer containing 150 mM or 200 mM NaCl led to the identification of a protein containing a WD40 domain (NCU09302) (Fig 5A). BLAST searches using the NCU09302 protein sequence identified yeast Cmr1 (Changed Mutation Rate 1; NP_010125.1) and human WDR76 (NP_079184.2) as putative homologs. Yeast Cmr1 and mammalian WDR76 localize to a sub-nuclear compartment in response to DNA damage, and these structures were shown to be distinct from DSB repair foci [75, 76]. To confirm the interaction between MUS-30 and NCU09302, we constructed a FLAG-tagged version of NCU09302 and performed FLAG affinity purifications in buffer containing 200 mM NaCl and in buffer containing 250 mM KCl. Analysis of both purified fractions by mass spectrometry identified MUS-30, confirming that NCU09302 and MUS-30 interact in vivo. We refer to NCU09302 as WDR76 based on similarity to the mammalian protein. We note that core histones were identified following purification of both MUS-30-3XFLAG and WDR76-3XFLAG, consistent with the predicted role of MUS-30 as a chromatin remodeling enzyme; however, these proteins were removed by our filter because they were also identified by purification of DCDC.

We performed several experiments to examine the functional role of the WDR76-MUS-30 interaction. We first asked if WDR76-3XFLAG is activated by phosphorylation in response to DNA damage. WDR76-3XFLAG was resolved on a Phos-tag gel and Western blots were performed using anti-FLAG antibodies. No change in mobility was observed, indicating that WDR76 is not phosphorylated in response to MMS-induced DNA damage (S4A Fig). We also examined phosphorylation of MUS-30-3XFLAG in the *Δwdr76* background and observed no change in MUS-30-3XFLAG phosphorylation (S4B Fig). We next asked if WDR76 associated with chromatin and if chromatin association was altered when MUS-30 was absent. We isolated the soluble and chromatin fraction from the *wdr76-3xflag* and *wdr76-3xflag;* Δ*mus-30* strains grown in the absence or presence of MMS. WDR76-3XFLAG was detected in both the soluble and chromatin fractions in the wildtype and the Δ*mus-30* background (S4C Fig). Chromatin association was not affected by DNA damage. We performed a similar experiment to examine chromatin association of MUS-30-3XFLAG in the wildtype and Δ*wdr76* strain background (S4D Fig). In both the presence and absence of DNA damage, MUS-30-3XFLAG was detected in the soluble and chromatin fractions in both strains. These data suggest that WDR76 and MUS-30 do not depend on one another in order to associate with chromatin. Finally, we asked if WDR76 impacted DSB repair by transforming a *mtr*::*basta* cassette in the *Δwdr76* and *Δmus-30*; *Δwdr76* double mutants (Table 1). In both strains the relative frequency of integration by HR or NHEJ and the transformation efficiency was similar to wildtype.

To confirm that interaction between MUS-30 and WDR76 is functionally important in vivo, we crossed *Δmus-30* to *Δwdr76* and tested for genetic interactions. The growth phenotype and the level of MMS-sensitivity were examined for wildtype, single mutant, and double mutant progeny. Both Δ*wdr76* and Δ*wdr76*; Δ*mus-30* strains displayed wildtype growth on minimal medium, similar to Δ*mus-30*. Notably, a positive genetic interaction was observed for Δ*mus-30* and Δ*wdr76* in the presence of MMS (Fig 5B and 5C), confirming that the two gene products interact functionally.

## Discussion

Chromatin remodelers can impact genome maintenance by regulating specific types of DNA repair, facilitating DNA replication, and enhancing propagation of DNA damage signals [3]. LSH/DDM1 homologs have been implicated in genome maintenance from yeast to humans, but how these proteins contribute to genome maintenance is not understood. Plant *ddm1* mutants are hypersensitive γ-radiation, UV-light, and MMS [34, 35]. Similarly, *Lsh*^-/-^ cells are hypersensitive to a number of DNA damaging agents and display muted induction of γH2A.X as well as diminished recruitment of γH2A.X-binding proteins following DNA damage. Based on these observations, it was concluded that LSH promotes efficient DSB repair. [36]. Our study provides additional evidence that LSH/DDM1 proteins are key regulators of genome stability and provides new insights into the role of an LSH/DDM1 family member in genome maintenance.

We propose that *N*. *crassa* MUS-30 plays an important role in preventing genome instability when replication forks encounter toxic base excision repair intermediates. This idea is supported by our findings that: 1) deletion of *mag-1* can partially rescue the MMS-sensitivity of Δ*mus-30* strains, 2) Δ*mus-30* and Δ*mei-3* interact genetically, 3) MUS-30 is not required for normal BER or DSB repair, and 4) MUS-30 interacts with WDR76. It is not known if other LSH/DDM1 enzymes in other systems act to maintain genome stability independently of DSB repair, but data from yeast and animals are compatible with the idea. A high throughput study in yeast found that *irc5*Δ strains accumulate spontaneous Rad52-GFP foci in the absence of exogenous DNA damage and exhibit elevated recombination rates with non-sister chromatids [37]. In animals, *Lsh* expression is highest in proliferating tissues and was correlated with the onset of S-phase [7–9, 13]. Moreover, Burrage and colleagues showed that DNA damage in LSH-deficient cells triggers normal cell cycle arrest, followed by rapid cell death once S-phase resumes [36]. Thus, it is possible that mammalian LSH and yeast Irc5 function during S-phase to prevent collapsed replication forks at specific types of DNA lesions.

Our protein interaction studies provide additional evidence supporting a conserved role for LSH/DDM1 in different systems. We found that *N*. *crassa* MUS-30 interacts with a well conserved protein, WDR76. Not only is the WDR76 protein conserved in fungi and animals, its interaction with LSH/DDM1 family members appears to be conserved across species. Proteomic analysis of Cmr1, the yeast WDR76 homolog, identified Irc5p as a putative Cmr1-interacting protein [77]. In addition, while this manuscript was in preparation, it was reported that mammalian LSH co-purifies with WDR76 [76]. Our observation that *mus-30* and *wdr76* interact genetically provides compelling evidence that physical interaction of MUS-30 and WDR76 is functionally important. Although the specific functions of WDR76 and its homologs are unknown, it was recently reported that both Cmr1 and mammalian WDR76 form DNA damage-dependent foci that are distinct from DSB repair centers [75, 76]. Thus, the interaction between WDR76 and MUS-30 provides additional evidence that MUS-30 is not directly involved in DSB repair. Interestingly, in the presence of the replication inhibitor HU, *cmr1Δ* exhibits positive genetic interactions with gene deletions of replication fork protection components [76]. We found a similar positive genetic interaction between *mus-30* and *wdr76* in the presence of MMS. These data could indicate that WDR76 somehow acts to destabilize stalled replication forks. Yeast Cmr1 localizes to a unique sub-nuclear compartment that was hypothesized to promote protein degradation, consistent with this possibility [76]. Alternatively, it was proposed that yeast Cmr1 negatively regulates the DNA damage response [76]. WDR76 may target MUS-30 and other components of the DNA damage response for degradation. Increased activity of other DNA repair components in the Δ*wdr76* strain could explain why the Δ*mus-30* phenotype is rescued by the *wdr76* deletion.

In plants and animals, LSH and DDM1 proteins have been extensively investigated for their role in regulating DNA methylation. It was suggested that changes in 5mC may lead to differential expression of DNA repair genes in *A*. *thaliana ddm1* mutants [35]. In contrast, it was proposed that mammalian LSH controls DNA repair and DNA methylation through distinct mechanisms [36]. Indeed, knock down of *Lsh*-knockdown caused hypersensitivity to DNA damage prior to methylation loss, demonstrating that loss of DNA methylation is not indirectly responsible for the hypersensitivity to DNA damage. Here, we found normal DNA methylation levels in *mus-30* strains by comprehensive MethylC-seq, clearly demonstrating that loss of 5mC does not drive DNA damage-sensitivity in Δ*mus-30* strains. It remains possible, however, that loss of DNA methylation in LSH/DDM1-deficient cells results in part from defective DNA repair functions. Future work is needed to fully understand how LSH/DDM1 family members function to regulate DNA methylation and contribute to genome stability.

## Materials and Methods

### Strains, growth media, and molecular analyses

All Neurospora strains used in this study are listed in S3 Table. Knockout strains were generated by the Neurospora gene knockout consortium [78] and obtained from the Fungal Genetics Stock Center [79]. Strains were grown at 32°C in Vogel's minimal medium (VMM) + 1.5% sucrose. Crosses were performed on modified synthetic cross medium [80]. For plating assays, Neurospora conidia were plated on VMM with 2.0% sorbose, 0.5% fructose, and 0.5% glucose. When relevant, plates included 200 μg/mL hygromycin or 400 μg/mL basta [81] or DNA damaging agents at the indicated concentration. For MMS survival curves, 200 cells were plated on minimal medium and on plates with increasing concentrations of MMS. The number of colonies was counted for each plate and plotted as a percentage of the no MMS control. At least two independent strain isolates were used for each concentration of MMS and at least three independent plating assays were performed to determine the average percent viability. Error bars depict standard deviation from the mean. Neurospora transformation [82], DNA isolation [83], protein isolation, and Western blotting [84] were performed as previously described. We performed affinity purification using M2 FLAG affinity gel (cat # A2220; Sigma-Aldrich). To separate soluble nuclear proteins from the chromatin fraction, cells were grown overnight and either left untreated or exposed to 0.015% MMS for three hours. Cells were then collected, ground in liquid Nitrogen, and resuspended in 1 mL of low salt extraction buffer (50mM HEPES-KOH pH 7.5, 150mM NaCl, 2mM EDTA, plus protease inhibitor tablets (Roche, Indianapolis, IN)). Extracts were centrifuged at 14,000 rpm and the supernatant containing soluble proteins was saved. The pellet was resuspended in 1mL of high salt extraction buffer (50mM HEPES-KOH pH 7.5, 600mM NaCl, 2mM EDTA, plus protease inhibitor tablets (Roche, Indianapolis, IN)) and subjected to sonication. Extracts were centrifuged at 14,000 rpm in a microfuge and the supernatant containing was saved as the chromatin fraction.

Protein identification by mass spectrometry was performed at the Oregon Health Sciences University proteomics core facility (Dr. Larry David) as described previously [85] except for the following modifications. Extraction Buffer (50 mM HEPES-KOH pH 7.6, 2 mM EDTA, 10% glycerol, 2 mM DTT, protease inhibitor cocktail (Roche, Indianapolis, IN)) contained either 150 mM NaCl, 200 mM NaCl, or 250 mM KCl as indicated. Total immunoprecipitated protein was run into an SDS-PAGE gel and a single band containing all immunoprecipitated proteins was excised, subjected to in-gel trypsin digestion, and analyzed on a Thermo LTQ Velos Pro linear ion trap instrument. 3X-FLAG and–GFP knock-in constructs were made by introduction of linear DNA fragments constructed by overlapping PCR using described plasmid vectors [54]. All primers, including primers for generating knock-in constructs and for amplifying and sequencing the NCU06306 gene from the *mus-30* strain, are listed in S4 Table. To analyze protein phosphorylation, FLAG-immunoprecipitated proteins were resolved on a modified 5% acrylamide gel containing 25 μM Phos-Tag (cat # 304–93526, Waka Pure Chemical Industries) before Western blotting [56].

### BER assay

Cells were grown for 11 hours in liquid VMM prior to addition of MMS to a final concentration of 0.035% MMS. After three hours, cells were collected using Buchner funnel and washed with 500mL of liquid VMM to remove MMS. Washed cells transferred to pre-warmed VMM and allowed to recover for 4 hr. Aliquots of cells were harvested and immediately frozen in liquid nitrogen prior to MMS treatment, and hourly during and after the 3 hours MMS treatment. Genomic DNA was isolated and 300 ng was digested by AP endonuclease (cat # M0282S, New England Biolabs), human alkyladenine DNA Glycosylase (cat # M0313S, New England Biolabs), or both enzymes for 1hr and 15min at 37C. Reactions were stopped by adding alkaline DNA loading buffer (50 mM NaOH, 1 mM EDTA, 3% Ficoll). Samples were resolved on a 1.2% alkaline agarose gel (1.5 M NaOH, 50 mM EDTA). Agarose gels were run in the cold room at 25V for 17hrs and then incubated in neutralization buffer (1.5 M NaCl, 1 M Tris-Cl pH 7.6) for 45 minutes before being stained with SYBR Gold (cat # S-11494, Life Technologies) for 40min and de-stained for 30 min before imaging.

### methylC-sequencing and data analysis

MethylC-seq libraries were prepared according to the following protocol [86]. Illumina sequencing was performed using an Illumina NextSeq500 Instrument at the University of Georgia Genomics Facility. Raw data were trimmed for adapters, preprocessed to remove low quality reads and aligned to the *N*. *crassa* (version 12) reference genome as previously described in [87]. Mitochondria sequence (which is fully unmethylated) was used as a control to calculate the sodium bisulfite reaction non-conversion rate of unmodified cytosines. Binomial test coupled with Benjamini-Hochberg correction was adopted to determine the methylation status of each cytosine. Identification of DMRs (Differentially Methylated Regions) was performed as described in [88]. Methylated regions in wild type was generated by running DMR finding between two wild type samples and an artificially created sample, which has 60X genome coverage but without any methylated cytosines. The maximum physical distance to combine two DMSs (Differential Methylated Sites) was set to 1kb. DMRs with at least 10 DMSs were reported and used for subsequent analyses. For metaplots, both upstream and downstream regions were divided into 20 bins each of 50bp in length for a total 1kb in each direction. Methylated regions were separated every 5%, for a total of 20 bins. Weighted methylation levels were computed for each bin as described previously[89].

Illumina sequence reads have been deposited into the NCBI GEO database (Accession #GSE70518).

## Supporting Information

S1 Fig Complementation of *mus-30* with *ncu06306*. The original *mus-30* strain is complemented by co-transformation of *bar* and *ncu06306****/****crf5-1*+. *mus-30* was co-transformed linear *bar* and *ncu06306****/****crf5-1*+ fragments or with *bar* alone, as indicated. Basta-resistant transformants were isolated, and spores from 40 individual transformants were spotted on plates containing VMM, basta, or MMS as indicated. (1) wildtype, (2) Δ*crf5-1* from the Neurospora knockout collection, (3) and Δ*dim-5* control strains are enclosed in the red box.
(TIF)

S2 Fig MUS-30 knock-in constructs are functional. Homozygous *mus-30-10xgly-gfp* and *mus-30-10xgly-3xflag* are able to grow in the presence of MMS.
(TIF)

S3 Fig Δ*mag-1* does not suppress Δ*mus-30* hypersensitivity to oxidative damage. Serial dilutions of conidia (10^4^−10^1^) were spot tested on minimal medium (VMM) with or without 100 μM tert-Butyl hydroperoxide for wildtype, Δ*mus-30*, Δ*mag-1*, *and* Δ*mus-30;* Δ*mag-1*.
(TIF)

S4 Fig WDR76 and MUS-30 do not depend on one another for phosphorylation or chromatin association. (A-B) Protein extracts were isolated from cells grown in the presence or absence of MMS (+ or -) and FLAG-tagged proteins were immunoprecipitated before incubation with or without lambda phosphatase (+ or—PPase). Proteins were resolved on a Phos-tag or SDS-PAGE gel as indicated, transferred to a membrane, and probed with an anti-FLAG antibody. (C-D) Soluble (Sol) and chromatin-bound (Chr) proteins were extracted from the indicated strains grown in the presence of absence of MMS (+ or -) and Western blots were probed with anti-FLAG antibodies. In D, blots were probed with an anti-H3 antibody to demonstrate successful separation of soluble and chromatin proteins.
(TIF)

S1 Table Methylation levels in wildtype and Δ*crf5-1* strains. (XLSX)

S2 Table Differential methylation analysis of wildtype and Δ*crf5-1* strains. (XLSX)

S3 Table Strains used in this study. (DOCX)

S4 Table Oligos used in this study. (DOCX)
