# Supplementary figures and images for: The LSH/DDM1 Homolog MUS-30 Is Required for Genome Stability, but Not for DNA Methylation in Neurospora crassa
Source: PLoS Genet. 2016 Jan 15;12(1):e1005790. doi: 10.1371/journal.pgen.1005790 (PMC4714748; doi:10.1371/journal.pgen.1005790)

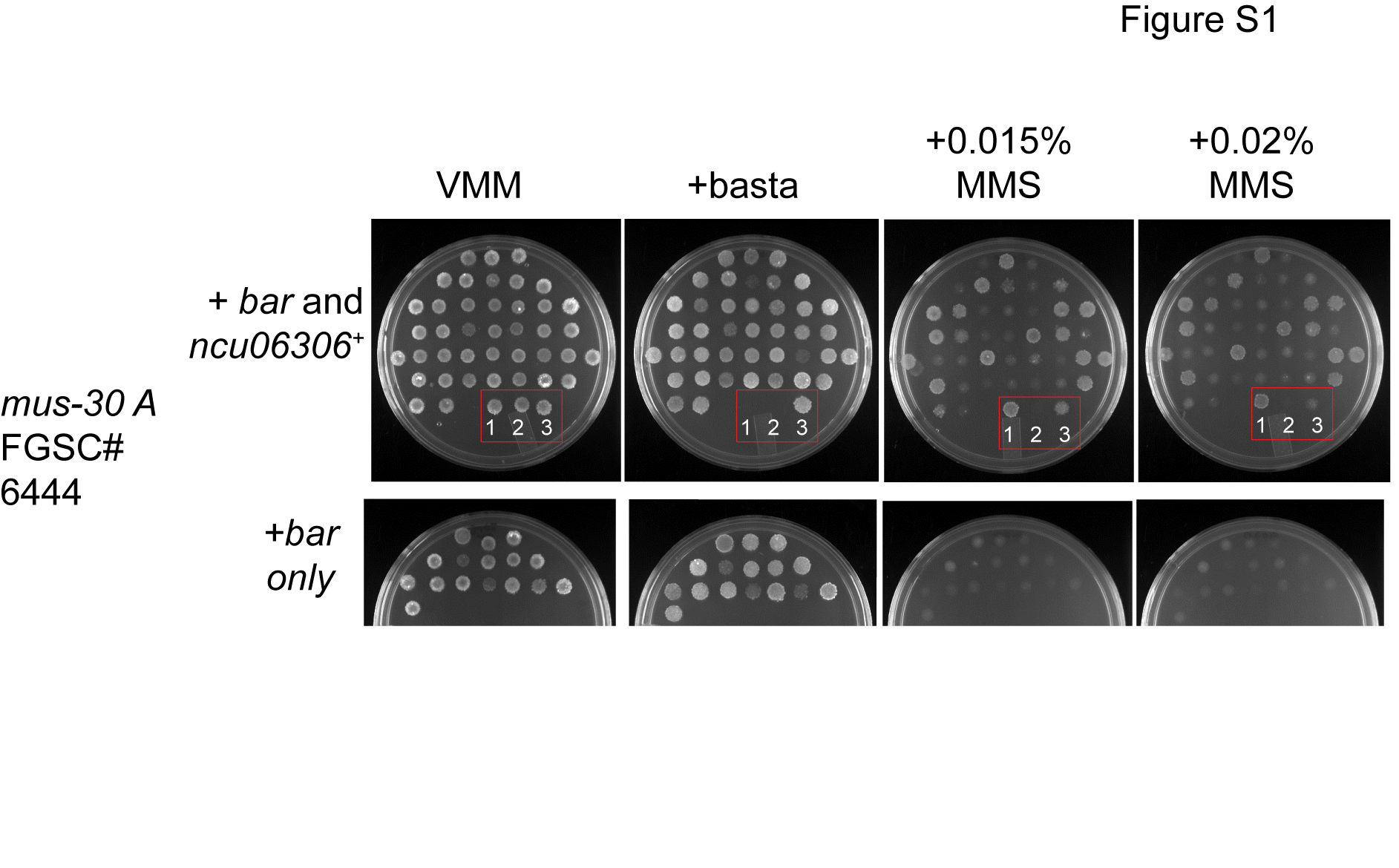

Supplement: S1 Fig — The original mus-30 strain is complemented by co-transformation of bar and ncu06306/crf5-1+. mus-30 was co-transformed linear bar and ncu06306/crf5-1+ fragments or with bar alone, as indicated. Basta-resistant transformants were isolated, and spores from 40 individual transformants were spotted on plates containing VMM, basta, or MMS as indicated. (1) wildtype, (2) Δcrf5-1 from the Neurospora knockout collection, (3) and Δdim-5 control strains are enclosed in the red box. (TIF) [file pgen.1005790.s001.tif]

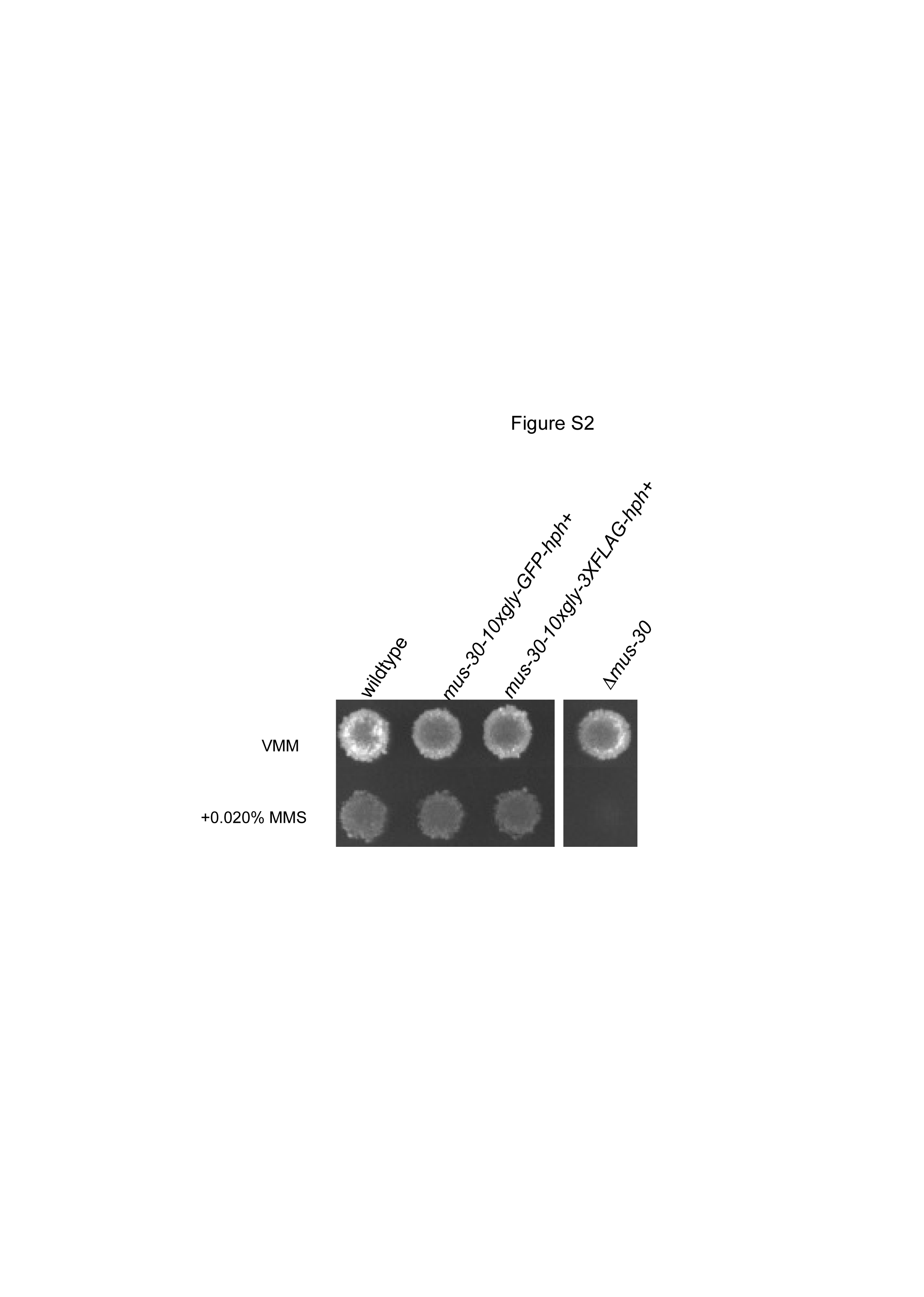

Supplement: S2 Fig — Homozygous mus-30-10xgly-gfp and mus-30-10xgly-3xflag are able to grow in the presence of MMS. (TIF) [file pgen.1005790.s002.tif]

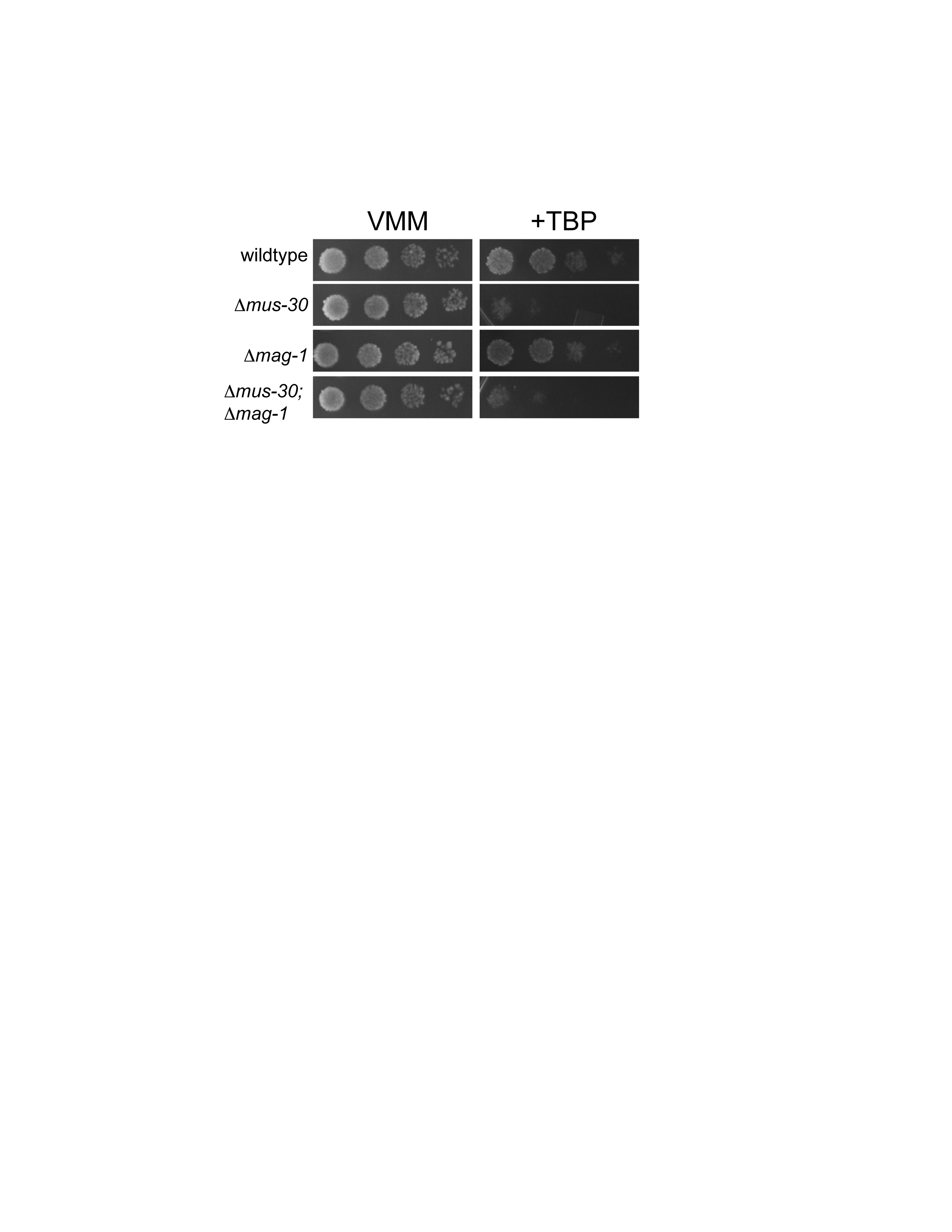

Supplement: S3 Fig — Serial dilutions of conidia (104−101) were spot tested on minimal medium (VMM) with or without 100 μM tert-Butyl hydroperoxide for wildtype, Δmus-30, Δmag-1, and Δmus-30; Δmag-1. (TIF) [file pgen.1005790.s003.tif]

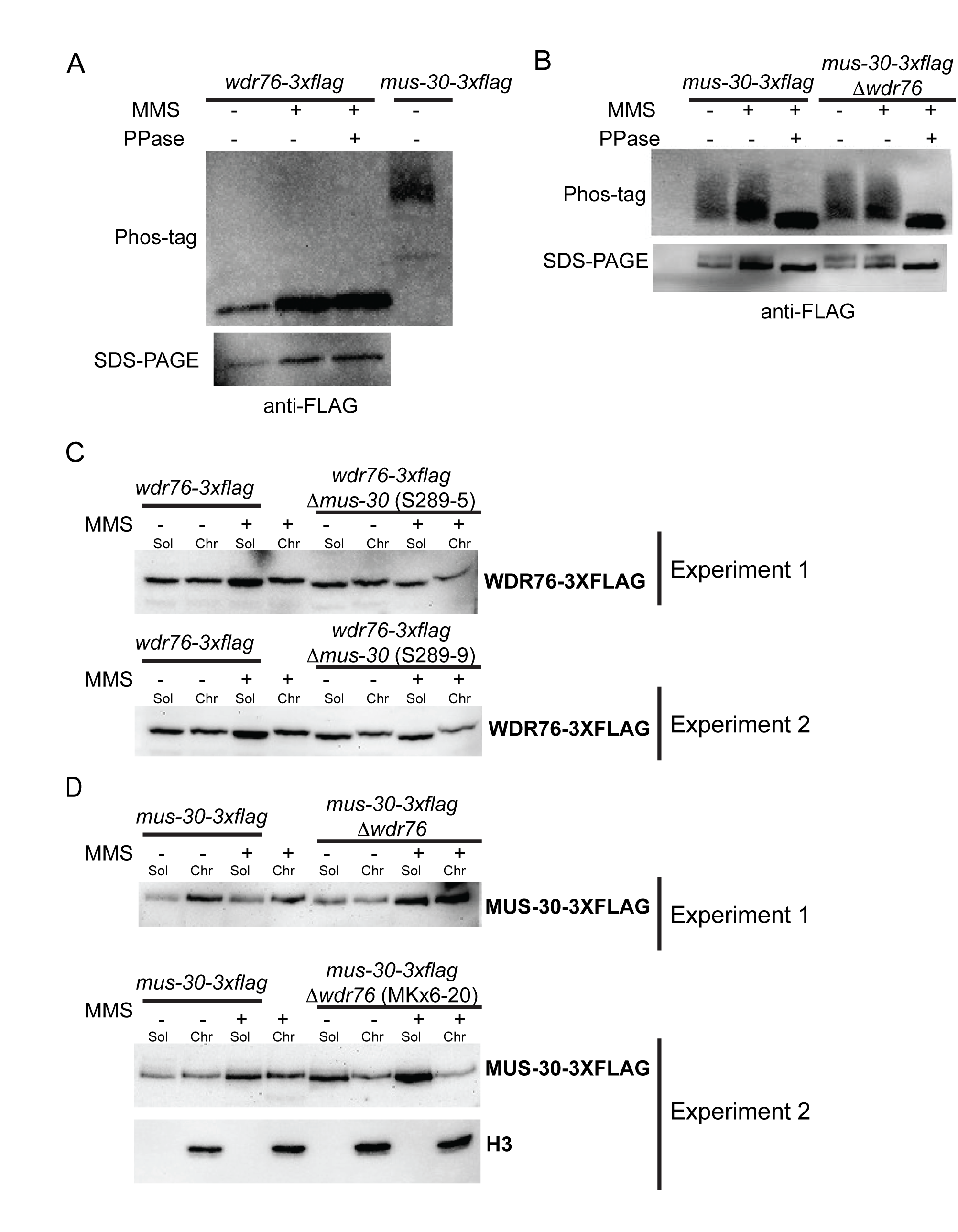

Supplement: S4 Fig — (A-B) Protein extracts were isolated from cells grown in the presence or absence of MMS (+ or -) and FLAG-tagged proteins were immunoprecipitated before incubation with or without lambda phosphatase (+ or—PPase). Proteins were resolved on a Phos-tag or SDS-PAGE gel as indicated, transferred to a membrane, and probed with an anti-FLAG antibody. (C-D) Soluble (Sol) and chromatin-bound (Chr) proteins were extracted from the indicated strains grown in the presence of absence of MMS (+ or -) and Western blots were probed with anti-FLAG antibodies. In D, blots were probed with an anti-H3 antibody to demonstrate successful separation of soluble and chromatin proteins. (TIF) [file pgen.1005790.s004.tif]
