# Supplementary material for: The LSH/DDM1 Homolog MUS-30 Is Required for Genome Stability, but Not for DNA Methylation in Neurospora crassa
Source: PLoS Genet. 2016 Jan 15;12(1):e1005790. doi: 10.1371/journal.pgen.1005790 (PMC4714748; doi:10.1371/journal.pgen.1005790)
Supplement: S3 Table — (DOCX) [file pgen.1005790.s007.docx]

| **Table S1. Strains used in this study** | | | |
| --- | --- | --- | --- |
| **Genotype** | **Strain #** | **Additional information** | **Reference** |
| Wildtype *(a)* | S1 | FGSC4200 | (1) |
| Wildtype (A) | S2 | FGSC2489 | (1) |
| *mus-52::Bar^+^* (a) | S8 |  | (1) |
| *Δcrf5/mus-30::Hph^+^* (a) | S281 | FGSC11758 | (1) |
| *Δcrf5/mus-30::Hph^+^* (A) | S282 | FGSC11759 | (1) |
| *mus-30-GFP::Hph^+^* | S425 | cross *mus-52::Bar^+^; crf5^+^::GFP::Hph^+^* x S2 | This Study |
| *mus-30-3XFLAG::Hph^+^* | S283 | cross *mus-52::Bar^+^; crf5^+^::3XFLAG::Hph^+^* x S2 | This Study |
| *Δmei-3::Hph^+^* | S480 | FGSC12433 | (1) |
| *Δwdr76::Hph^+^* (heterkaryon) ; *Δmus-52::Bar^+^* | S481 | FGSC22674 |  |
| *Δwdr76::Hph^+^* | S492 | cross of *Sad-1 A* x FGSC22674 | (1) |
| *Δmag-1::Hph^+^* | S482 | FGSC18428 | (1) |
| Wildtype | S452 | cross FGSC11759 x FGSC18428 | This study |
| Wildtype | S453 | cross FGSC11759 x FGSC18428 | This study |
| *Δmus-30::Hph^+^ Δmag-1::Hph^+^* | S454 | cross FGSC11759 x FGSC18428 | This study |
| *Δmus-30::Hph^+^ Δmag-1::Hph^+^* | S455 | cross FGSC11759 x FGSC18428 | This study |
| *Δmag-1::Hph^+^* | S456 | cross FGSC11759 x FGSC18428 | This study |
| *Δmag-1::Hph^+^* | S457 | cross FGSC11759 x FGSC18428 | This study |
| *Δmag-1::Hph^+^* | S458 | cross FGSC11759 x FGSC18428 | This study |
| *Δmag-1::Hph^+^* | S459 | cross FGSC11759 x FGSC18428 | This study |
| *Δmus-30::Hph^+^* | S460 | cross FGSC11759 x FGSC18428 | This study |
| *Δmus-30::Hph^+^* | S461 | cross FGSC11759 x FGSC18428 | This study |
| *Δmus-30::Hph^+^* | S462 | cross FGSC11759 x FGSC18428 | This study |
| Wildtype | S463 | cross FGSC11759 x FGSC12433 | This study |
| Wildtype | S464 | cross FGSC11759 x FGSC12433 | This study |
| *Δmus-30::Hph^+^* | S465 | cross FGSC11759 x FGSC12433 | This study |
| *Δmus-30::Hph^+^* | S466 | cross FGSC11759 x FGSC12433 | This study |
| *Δmei-3::Hph^+^* | S467 | cross FGSC11759 x FGSC12433 | This study |
| *Δmei-3::Hph^+^* | S468 | cross FGSC11759 x FGSC12433 | This study |
| *Δmus-30::Hph^+^ Δmei-3::Hph^+^* | S469 | cross FGSC11759 x FGSC12433 | This study |
| *Δmus-30::Hph^+^ Δmei-3::Hph^+^* | S470 | cross FGSC11759 x FGSC12433 | This study |
| *Δmus-30::Hph^+^ Δmei-3::Hph^+^* | S471 | cross FGSC11759 x FGSC12433 | This study |
| Wildtype | S472 | cross FGSC11759 x FGSC22674 | This study |
| Wildtype | S473 | cross FGSC11759 x FGSC22674 | This study |
| *Δmus-30::Hph^+^* | S474 | cross FGSC11759 x FGSC22674 | This study |
| *Δmus-30::Hph^+^* | S475 | cross FGSC11759 x FGSC22674 | This study |
| *Δwdr76::Hph^+^* | S476 | cross FGSC11759 x S492 | This study |
| *Δwdr76::Hph^+^* | S477 | cross FGSC11759 x S492 | This study |
| *Δmus-30::Hph^+^ Δwdr76::Hph^+^* | S478 | cross FGSC11759 x S492 | This study |
| *Δmus-30::Hph^+^ Δwdr76::Hph^+^* | S479 | cross FGSC11759 x S492 | This study |
| *mus-30^FK115^* (A) | S338 | FGSC6444 | (2) |
| *mus-30 ^FK115^* (a) | S339 | FGSC6445 | (2) |
| (*mus-30 ^FK115^; ::Bar^+^* TF1 *+* (*ΔNCU06306::Hph^+^*) | S483 | heterokaryon | This study |
| (*mus-30 ^FK115^; ::Bar^+^* TF2 *+* (*ΔNCU06306::Hph^+^)* | S484 | heterokaryon | This study |
| (*mus-30 ^FK115^; ::Bar^+^* TF3 *+* (*ΔNCU06306::Hph^+^*) | S485 | heterokaryon | This study |
| (*mus-30 ^FK115^; ::Bar^+^* TF4*+* (*ΔNCU06306::Hph^+^*) | S486 | heterokaryon | This study |
| (*mus-30 ^FK115^; ::Bar^+^* TF5 *+* (*ΔNCU06306::Hph^+^*) | S487 | heterokaryon | This study |
| (*ΔNCU06306::Hph^+^* + *Δdim-5::Bar^+^*) | S488 | heterokaryon | This study |
| (*ΔNCU06306::Hph^+^* + *Δdim-5::Bar^+^*) | S489 | heterokaryon | This study |
| (*ΔNCU06306::Hph^+^* + *Δdim-5::Bar^+^*) | S490 | heterokaryon | This study |
| *mus-30 ^FK115^; :: Bar+; ::NCU06306^+^* | S340 | FGSC6444 transformed with wildtype NCU06306 | This study |
| *wdr76-3XFLAG::Hph+* | S491 | Cross of *mus-52::Bar^+^;*  *wdr76-3XFLAG::Hph+* x S2 | This study |
| *wdr76-3xflag;*  *Δmus-30* | S289-5 | Cross of S491 to S281 | This study |
| *wdr76-3xflag;*  *Δmus-30* | S289-9 | Cross of S491 to S281 | This study |
| *mus-30-3xflag*  *Δwdr76* | MKx6-5 | Cross of S283 to S476 | This study |
| *mus-30-3xflag*  *Δwdr76* | MKx6-20 | Cross of S283 to S476 | This study |

1. Colot, H.V., Park, G., Turner, G.E., Ringelberg, C., Crew, C.M., Litvinkova, L., Weiss, R.L., Borkovich, K.A. and Dunlap, J.C. (2006) A high-throughput gene knockout procedure for Neurospora reveals functions for multiple transcription factors. *Proc Natl Acad Sci U S A*, **103**, 10352-10357.

2. Kafer, E. and Luk, D. (1989) Sensitivity to bleomycin and hydrogen peroxide of DNA repair-defective mutants in Neurospora crassa. *Mutat. Res.*, **217**, 75-81.
