# Supplementary material for: The LSH/DDM1 Homolog MUS-30 Is Required for Genome Stability, but Not for DNA Methylation in Neurospora crassa
Source: PLoS Genet. 2016 Jan 15;12(1):e1005790. doi: 10.1371/journal.pgen.1005790 (PMC4714748; doi:10.1371/journal.pgen.1005790)
Supplement: S4 Table — (DOCX) [file pgen.1005790.s008.docx]

| **Table S4. Oligonucleotides used in this study** | |
| --- | --- |
| Oligo Name | Oligo Sequence |
| NCU06306 FP | ACG AACTGT CGC GAG AAG AC |
| NCU06306 RP | CGG CAT ACA ATT GAA ACGTG |
| NCU06306 CDS FP | GAG GTC GAC GGT ATC GAT AAG CTT GAT ATC GTC AAG ATC TTC CTG CTG TC |
| NCU06306 CDS RP | CCT CCG CCT CCG CCT CCG CCG CCT CCG CCC CTC TTT TTC TTC TCA CCC GC |
| NCU06306 UTR FP | TGC TAT ACG AAGTTATGG ATC CGA GCT CGT GTG GTG CTC GTG ATT GCCT |
| NCU06306 UTR RP | ACC GCG GTG GCG GCC GCT CTA GAA CTA GTT TGC CCGTGA TTA ATT CGT T |
| NCU09302 CDS FP | GAGGTCGACGGTATCGATAAGCTTGATATCATTTGGGGCTTAACTGACCAC |
| NCU09302 CDS RP | CCTCCGCCTCCGCCTCCGCCGCCTCCGCCCATCCATAAACACAATTTCCC |
| NCU09302 UTR FP | TATTCTATAGTGTCACCTAAATAGCTTGGTGGTGGCAGTGTTCATGTGTC |
| NCU09302 UTR RP | ACCGCGGTGGCGGCCGCTCTAGAACTAGTGTCCCTAGGCTACCTGATTGG |
| bar FP | CCGTCGACAGAAGATGATATTGAAGGAGC |
| bar RP | AATTAACCCTCACTAAAGGGAACAAAAGC |
| sequencing primer FP M M30 | GCCTTTGCCTTGTACCCATT |
| sequencing primer FP I M31 | AAAGGATCTGGACCGGCTTC |
| sequencing primer FP I M32 | CTCAAGGACCGAGCGAATCT |
| sequencing primer FP I M33 | AAGACCAGCGCACTTACTCT |
| sequencing primer FP I M34 | GAGCTACGTTCATGGGACG |
| sequencing primer FP I M35 | AACGGCGGGTGAGAAGAAAA |
| sequencing primer FPM M36 | TGGTGGTGAGAATTGGGTTG |
